# Guinea Pig Model for Evaluating the Potential Public Health Risk of Swine and Avian Influenza Viruses

**DOI:** 10.1371/journal.pone.0015537

**Published:** 2010-11-23

**Authors:** Yipeng Sun, Yuhai Bi, Juan Pu, Yanxin Hu, Jingjing Wang, Huijie Gao, Linqing Liu, Qi Xu, Yuanyuan Tan, Mengda Liu, Xin Guo, Hanchun Yang, Jinhua Liu

**Affiliations:** 1 Key Laboratory of Zoonosis of Ministry of Agriculture, College of Veterinary Medicine, China Agricultural University, Beijing, China; 2 The Shandong Animal Disease Control Center, Jinan, Shandong, China; 3 CAS Key Laboratory of Pathogenic Microbiology and Immunology Institute of Microbiology, Chinese Academy of Sciences, Beijing, China; University of Hong Kong, Hong Kong

## Abstract

**Background:**

The influenza viruses circulating in animals sporadically transmit to humans and pose pandemic threats. Animal models to evaluate the potential public health risk potential of these viruses are needed.

**Methodology/Principal Findings:**

We investigated the guinea pig as a mammalian model for the study of the replication and transmission characteristics of selected swine H1N1, H1N2, H3N2 and avian H9N2 influenza viruses, compared to those of pandemic (H1N1) 2009 and seasonal human H1N1, H3N2 influenza viruses. The swine and avian influenza viruses investigated were restricted to the respiratory system of guinea pigs and shed at high titers in nasal tracts without prior adaptation, similar to human strains. None of the swine and avian influenza viruses showed transmissibility among guinea pigs; in contrast, pandemic (H1N1) 2009 virus transmitted from infected guinea pigs to all animals and seasonal human influenza viruses could also horizontally transmit in guinea pigs. The analysis of the receptor distribution in the guinea pig respiratory tissues by lectin histochemistry indicated that both SAα2,3-Gal and SAα2,6-Gal receptors widely presented in the nasal tract and the trachea, while SAα2,3-Gal receptor was the main receptor in the lung.

**Conclusions/Significance:**

We propose that the guinea pig could serve as a useful mammalian model to evaluate the potential public health threat of swine and avian influenza viruses.

## Introduction

Five influenza pandemics have resulted in significant morbidity and mortality around the world. Genetic analyses revealed that these pandemic strains were entirely or partially derived from the viruses of animal origin, including swine and avian strains [1]. So far, animal influenza viruses have repeatedly transmitted to humans with increasing genetic diversity, such as avian H5N1 and H9N2 influenza viruses, and swine H1N1, H1N2, H3N2 influenza viruses [2], [3], which served as an important reminder that another influenza pandemic is highly likely. Among these swine and avian influenza viruses, in addition to highly pathogenic H5N1 avian influenza viruses, many low pathogenic viruses are also high on the list of candidates that could potentially cause the next human pandemic. The efficiency of transmission is a key factor in determining the severity of influenza epidemics. Despite evidence for limited human-to-human transmission of some animal influenza viruses, they have yet to exhibit sustained transmission among humans [4], [5]. However, with typical human flu-like illness in humans [6], [7] that may be unrecognized, these less pathogenic animal viruses can develop the ability of human-to-human transmission. Thus, a mammalian disease model is needed to evaluate the epidemic potential of these viruses.

Mice are a common mammalian model used in influenza research but are not ideal for transmission studies and even the 1918 pandemic strain could not transmit between mice [8]. Ferrets, however, are susceptible to influenza infection and develop similar symptoms to humans and effectively transmit between individuals. Nevertheless, the ferret model presents several practical disadvantages [9]. Recently, guinea pigs have been shown to be an acceptable alternative mammalian model for the study of human influenza A virus transmission [10]–[12]. Guinea pigs are an attractive model host due to their high susceptibility to and ability to transmit low-passage human isolates (in contrast to mice), and based on several practical considerations such as size and cost, make them considerably more convenient for research purposes than ferrets [8]. Additionally, guinea pigs were also proposed as a useful model to understand the virus-host interactions in influenza A virus infection [13] because they possess similar innervations in the airway to humans [14] and also have human-like bronchus-associated lymphoid tissue in lungs [15].

Host receptor expression is one of the important factors influence the infection of the respiratory tracts by influenza viruses. The HA protein of influenza viruses initiates infection by binding sialic acid (SA) which are bound to glycans through an α2,3 or α2,6 linkage [16]. In general, human influenza viruses have a binding preference for SAα2,6-Gal and avian influenza viruses preferentially binding SAα2,3-Gal [17], [18]. The distribution of these two forms of sialic acid compounds is known to be cell and species specific. Thus, host receptor distribution is an important factor in consideration when evaluating an animal model.

Thus far, influenza studies using the guinea pig model have been performed with 1918 pandemic [19], 2009 pandemic H1N1 [11], seasonal human H1N1 and H3N2 [10], [13], and avian H5N1 influenza viruses [15], [20], [21], but little is known about other swine and avian influenza virus infections, particularly those isolated in recent years. To evaluate the applicability of the guinea pig model for swine and avian influenza viruses, we describe the infectivity and transmissibility in guinea pigs of the subtypes of viruses that repeatedly transmit to humans (swine H1N1, H1N2, H3N2 and avian H9N2 influenza viruses) compared with 2009 pandemic and human seasonal influenza viruses. Additionally, the expression of SAα2,3-Gal and SAα2,6-Gal linked receptors in guinea pig respiratory tissues were determined.

## Results

### Replication of human, swine and avian influenza viruses in guinea pigs

**Figure 1:**
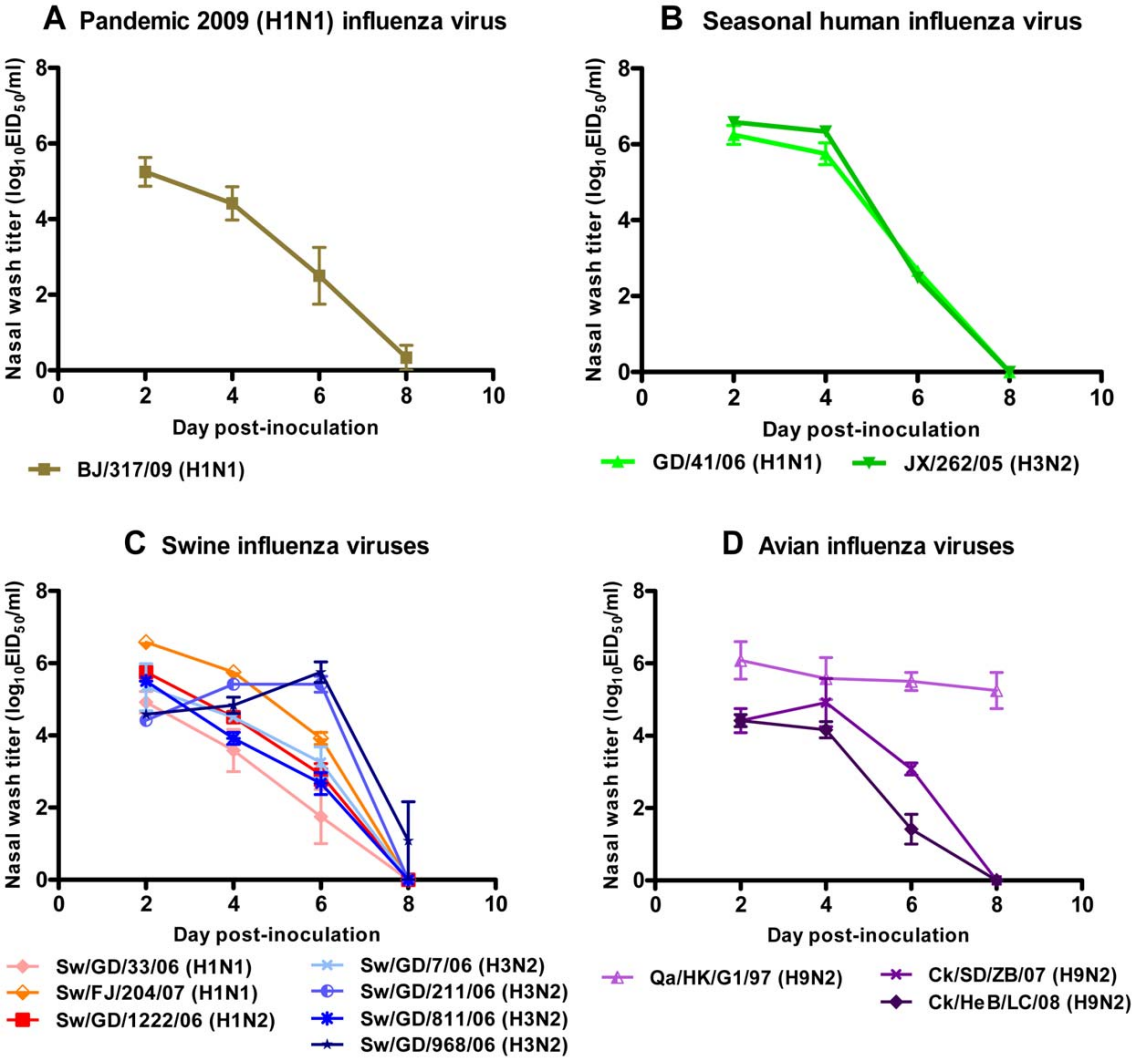
Growth kinetics of pandemic H1N1. (A), seasonal human (B), swine (C), and avian (D) influenza viruses in the nasal passages of guinea pigs. Groups of three guinea pigs were intranasally inoculated with 10^6^ EID_50_ of virus. Nasal wash titers (mean ± SD) are plotted on days 2, 4, 6, and 8 p.i. Nasal wash titers of different strains are represented by different colors. The lower limit of detection was 10^1.5^ EID_50_/mL.

To evaluate the replication of human, swine and avian influenza viruses in guinea pigs, groups of 15 animals were anesthetized and intranasally inoculated with 10^6^ 50% egg infective dose (EID_50_) of each virus listed in Table 1. On days 2, 4, 6, and 8 post-inoculation (p.i.), three animals from each group were anesthetized and the tracheas, lungs, hearts, livers, spleens, kidneys, brains, and colons were collected for virus titration. Nasal washes from the remaining three animals were collected on days 2, 4, 6, and 8 p.i., and titrated by EID_50_ assay. The study indicated that, similar to human influenza viruses, all swine and avian influenza viruses used in this study replicated efficiently in the nasal passages of guinea pigs, with peak virus titers in nasal washings ranging from 10^4.4^ to 10^6.6^ EID_50_/mL. The virus titer of BJ/317/09 (H1N1) was lower than those of other pandemic 2009 (H1N1) virus reported previously [22], possibly due to strain specificity. Most viruses grew to peak titers on day 2 p.i. in the nasal passages of inoculated guinea pigs and dropped to undetectable levels by day 8 p.i.. However, Sw/GD/211/06 (H3N2), Sw/GD/968/06 (H3N2) and Qa/HK/G1/97 (H9N2) strains possessed different shedding kinetics and were cleared more slowly from the guinea pig nasal passages than other viruses (Fig. 1 A–D). The nasal virus titers of these three virus-infected animals remained high (>10^5.0^ EID_50_/mL) on day 6 p.i.. In particular, the Qa/HK/G1/97 (H9N2) virus still replicated well in nasal passages on day 8 p.i. with mean titers of 10^5.3^ EID_50_/mL.

**Table 1:** Virus, clinical signs, virus replication, transmission, and seroconversion of the guinea pigs.

Virus name	Abbreviation	Host	Subtype	Phylogenetic lineage of HA gene	Inoculated guinea pigs			Contact guinea pigs		Transmission
					Sneezing (day of onset)	Peak mean nasal wash titer±SD (day)^b^	Seroconversion (HI titer range)^c^	Virus detected in nasal wash^d^	Seroconversion (HI titer range)^c^	
A/Beijing/317/09	BJ/317/09	Human	H1N1	Novel swine-origin	3/3^a^(2)	5.3±0.7 (2)	3/3 (160–320)	3/3	3/3 (40–160)	Efficient
A/Guangdong/41/06	GD/41/06	Human	H1N1	Human Seasonal	1/3 (3)	6.3±0.4 (2)	3/3 (320)	1/3	2/3 (80–160)	Inefficient
A/Jiangxi/262/05	JX/262/05	Human	H3N2	Human Seasonal	0/3	6.6±0.1 (2)	3/3 (160–320)	2/3	1/3 (80)	Inefficient
A/swine/Guangdong/33/06	Sw/GD/33/06	Swine	H1N1	Classical swine	0/3	4.9±0.5 (2)	3/3 (40)	0/3	0/3	None
A/swine/Fujian/204/07	Sw/FJ/204/07	Swine	H1N1	European avian-like swine	0/3	6.6±0.1 (2)	3/3 (40–80)	0/3	0/3	None
A/swine/Guangdong/1222/06	Sw/GD/1222/06	Swine	H1N2	North American triple	0/3	5.8±0.3 (2)	3/3 (80)	0/3	0/3	None
A/swine/Guangdong/7/06	Sw/GD/7/06	Swine	H3N2	Recent human-like	0/3	5.3±1.1 (2)	3/3 (40)	0/3	0/3	None
A/swine/Guangdong/211/06	Sw/GD/211/06	Swine	H3N2	Intermediate human-like	0/3	5.4±0.4 (6)	3/3 (40–80)	0/3	0/3	None
A/swine/Guangdong/811/06	Sw/GD/811/06	Swine	H3N2	Intermediate human-like	0/3	5.5±0.3 (2)	3/3 (40–80)	0/3	0/3	None
A/swine/Guangdong/968/06	Sw/GD/968/06	Swine	H3N2	Intermediate human-like	0/3	5.8±0.5 (6)	3/3 (40–80)	0/3	0/3	None
A/quail/Hong Kong/G1/97	Qa/HK/G1/97	Quail	H9N2	G1-like	0/3	6.1±0.5 (2)	3/3 (80)	0/3	0/3	None
A/chicken/Shandong/ZB/07	Ck/SD/ZB/07	Chicken	H9N2	Ck/Bei-like	0/3	4.9±1.2 (4)	3/3 (40–80)	0/3	0/3	None
A/chicken/Hebei/LC/08	Ck/HeB/LC/08	Chicken	H9N2	Ck/Bei-like	0/3	4.4±0.3 (2)	3/3 (40–80)	0/3	0/3	None

a Number of inoculated guinea pigs/total number.

b Peak nasal wash titers are expressed as the mean log_10_EID_50_/mL±SD.

c Serum was collected on day 21 p.i., and homologous strains were used with chicken RBCs in HI assays.

d The lower limit of detection was 10^1.5^ EID_50_/mL.

Compared to nasal passages, tracheas and lungs had much lower virus titers and viruses were cleared more quickly from these organs (Table 2). Two human seasonal influenza viruses were not detected in the lower respiratory tract, while the 2009 pandemic H1N1 virus could replicate in the lung. For animal influenza viruses, only the Sw/FJ/204/07 (H1N1), Sw/GD/1222/06 (H1N2), Qa/HK/G1/97 (H9N2) and Ck/SD/1/06 (H9N2) viruses could be detected in the lung. All of these viruses could not be detected in tracheas and lungs on day 8 p.i.. It is interesting that the European avian-like swine H1N1 (Sw/FJ/204/07) and North American triple H1N2 (Sw/GD/1222/06) viruses, that were proposed to be the progenitors of 2009 pandemic H1N1 influenza viruses, both replicated more efficiently and distributed in more tissues than other swine influenza viruses, similar to the 2009 pandemic H1N1 virus (BJ/317/09). No virus was detected in other organs, including heart, liver, spleen, kidney, brain and colon during the observation period. Taken together, the replication of tested influenza viruses was restricted to the respiratory system of guinea pigs and virus replication in the upper respiratory tract was higher than in the lower respiratory tracts. Similar to human strains, swine and avian influenza viruses replicated efficiently in nasal tracts without prior adaptation.

**Table 2:** Replication of influenza viruses in guinea pigs.

Virus (Subtype)	No. of positive guinea pigs/total no. of guinea pigs on dpi (mean virus titers [ log_10_EID_50_/gram]±SD)^a^					
	2		4		6	
	Trachea	Lung	Trachea	Lung	Trachea	Lung
BJ/317/09 (H1N1)	3/3(3.4±0.1)	3/3(3.7±0.1)	3/3(3.3±0.1)	2/3(2.1±1.6)	1/3(3.3)	0/3
GD/41/06 (H1N1)	3/3(3.5±0.3)	0/3	3/3(1.8±1.3)	0/3	0/3	0/3
JX/262/05 (H3N2)	3/3(3.6±0.3)	0/3	3/3(3.3±0.1)	0/3	1/3(<)	0/3
Sw/GD/33/06 (H1N1)	3/3(2.6±1.4)	0/3	3/3(2.4±1.2)	0/3	1/3(<)	0/3
Sw/FJ/204/07 (H1N1)	2/3(3.4±0.2)	2/3(3.4±0.2)	3/3(3.4±0.2)	2/3(3.3±0.1)	1/3(3)	1/3(3.3)
Sw/GD/1222/06 (H1N2)	2/3(3.4±0.2)	3/3(3.7±0.1)	1/3(3.0)	3/3(3.2±0.1)	1/3(<)	3/3(1.7±1.2)
Sw/GD/7/06 (H3N2)	2/3(3.5±0.4)	0/3	1/3(3.5)	0/3	1/3(3.0)	0/3
Sw/GD/211/06 (H3N2)	0/3	0/3	1/3(4.3)	0/3	1/3(4.0)	0/3
Sw/GD/811/06 (H3N2)	0/3	0/3	0/3	0/3	0/3	0/3
Sw/GD/968/06 (H3N2)	1/3(3.5)	0/3	2/3(2.3±1.8)	0/3	0/3	0/3
Qa/HK/G1/97 (H9N2)	3/3(4.0±0.7)	3/3(4.3±0.7)	0/3	0/3	0/3	0/3
Ck/SD/ZB/07 (H9N2)	1/3(3.3)	3/3(3.8±0.5)	1/3(3.3)	3/3(3.4±0.3)	0/3	0/3
Ck/HeB/LC/08 (H9N2)	1/3(<)	0/3	0/3	0/3	0/3	0/3

a <, virus was detected in the undiluted samples. The lower limit of detection was 10^1.5^ EID_50_/mL of tissue homogenate.

### Histopathology of human, swine, and avian influenza viruses in guinea pigs

To compare the histopathological changes in human, swine, and avian influenza viruses in guinea pigs, the nasal, trachea, and lung specimens from infected animals on day 4 p.i. were fixed in 10% neutral buffered formalin and processed for routine histology. The representative histopathology changes are shown in Fig. S1. A–L. Nasal lesions in infected guinea pig were characterized by phlebectasia and congestion in the submucosal capillaries and veins (Fig S1 A–C), dropout of the mucous epithelium and erythrocytes adhering to the surface of the mucosa (Fig S1 D). The tracheal mucous membrane showed edema and thickening (Fig S1 E) and dropout of tracheal epithelial cells (Fig S1 G, H). The infected lungs showed bronchiolitis with inflammatory cellular infiltrate and dropout of mucous epithelial cells in the bronchioles (Fig S1 I), peribronchiolitis was also observed with edema and inflammatory cells infiltrate around bronchioles and blood vessels (Fig S1 I, K). The results suggested that the tested viruses showed varying levels of pathological lesions. In particular, the histopathological changes in guinea pigs infected with Qa/HK/G1/97 were more severe than those caused by other viruses. In this group, the epithelial cells of the tracheal mucosa were severely desquamated resulting in exposure of the lamina propria (Fig S1 H). Severe bronchiolitis, peribronchiolitis, and bronchopneumonia were also observed with alveolar lumens and bronchioles flooded with edema fluid mixed with fibrin, erythrocytes, and inflammatory cells (Fig S1 L). For the tissues in virus was not detected, lesions were mild or absent (Fig S1 F, J).

### Transmission of human, swine, and avian influenza viruses in guinea pigs

**Figure 2:**
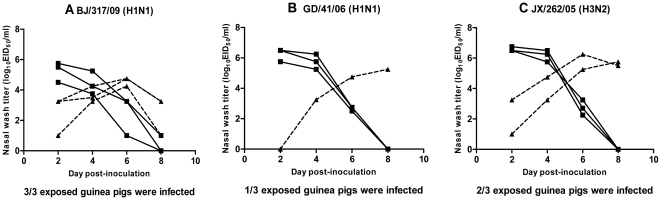
Contact transmission of pandemic H1N1 BJ/317/09, seasonal human H1N1 GD/41/06, and seasonal human H3N2 Jiangxi/262/05 in guinea pigs. Three guinea pigs were intranasally inoculated with 10^6^ EID_50_ of BJ/317/09 (A), GD/41/06 (B) or JX/262/05 (C) viruses and housed in a cage for each virus. At 24 h p.i., three naïve guinea pigs were placed in the same cage for each virus. Nasal washing titers are plotted as a function of time post-inoculation. Titers of intranasally inoculated animals are represented by dashed lines and filled squares; titers of exposed guinea pigs are shown with solid lines and filled triangles.

We next tested the propensity of the viruses to be transmitted by direct contact. Briefly, groups of three animals were intranasally inoculated with 10^6^ EID_50_ of tested virus and housed in a cage. At 24 h p.i., three naïve guinea pigs were placed in the same cage for each virus. On days 2, 4, 6, and 8 p.i., the nasal washings of the three inoculated animals and three contact animals were collected and titered by EID_50_ assay. Evidence of transmission was based on the detection of virus in the nasal washing and seroconversion at the end of the three-week observation period. All of the viruses replicated efficiently in inoculated animals, as for the replication study; however, the transmissibility of these viruses in guinea pigs was variable. As shown in Fig. 2 and Table 1, transmission of BJ/317/09 (H1N1) virus occurred with 100% efficiency: all three exposed animals were detected to be positive on day 3 post-exposure (corresponding to day 4 p.i.). The two seasonal human influenza viruses transmitted less efficiently with only one of three and two of three exposed animals becoming infected, respectively. However, no virus shedding or seroconversion was detected in any direct contact animal of swine and avian influenza viruses. These results demonstrate that although the swine and avian influenza viruses replicated effectively in the inoculated guinea pigs, they lacked successful direct contact transmissibility. As no contact transmission occurred for swine and avian influenza viruses, we did not test the propensity of influenza viruses to be transmitted *via* the aerosol route.

Of note, we observed that guinea pigs in the inoculated group and contact group of BJ/317/09 (H1N1) infections began to sneeze on days 3 and 4 p.i., respectively. Some guinea pigs in the GD/41/06 (H1N1) infected group also sneezed during the observation period. Except for sneezing, no other clinical abnormalities were observed in any of the animals during the observation period.

### Receptor distribution in the respiratory system of guinea pigs

**Figure 3:**
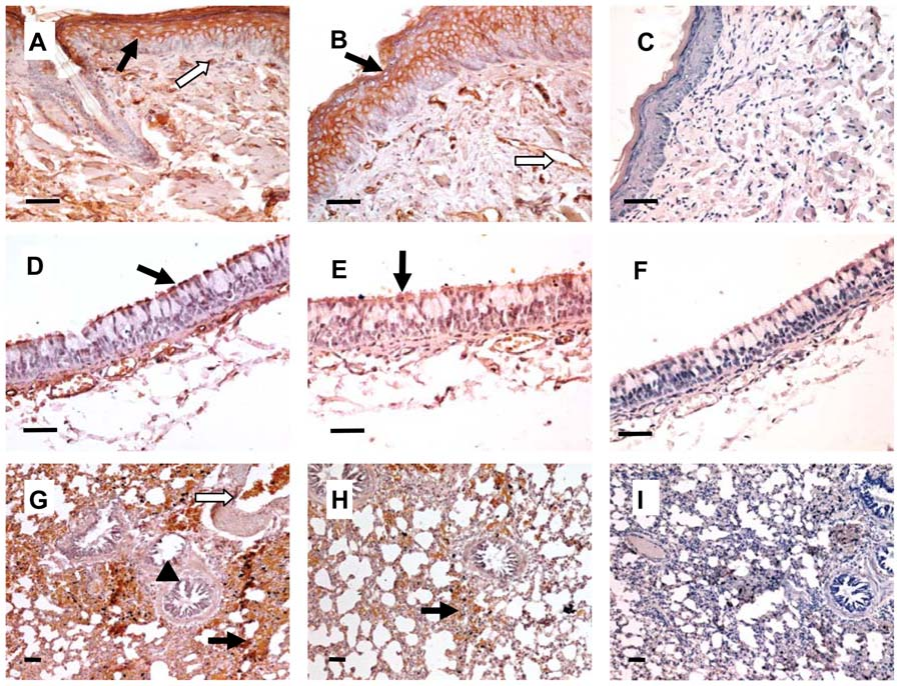
Receptor distribution in the respiratory system of guinea pigs. Tissue sections were stained with MAA II (specific for binding the α-2,3-linked sialic acid) (A, D and G) or SNA (specific for binding the α-2,6 linked sialic acid) (B, E and H). C, F, and I, neuraminidase pre-treatment was applied prior to lectin staining. After pretreatment with neuraminidase, neither MAA II nor SNA showed a positive reaction. This confirms the specificity of both lectin stainings. A–C, nasal mucosa. (A) MAA staining is visible on stratified squamous epithelial cells (↑) and vascular endothelial cells (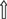). (B) SNA staining is identified on stratified squamous epithelial cells (↑) and vascular endothelial cells (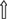). D–F, trachea. (D) MAA II staining is visible on almost all epithelial cells (↑). (E) SNA staining is visible on almost all epithelial cells (↑). G–I, lung. (*G*) MAA II staining is positive on alveolar cells (↑), vascular endothelial cells (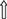) and bronchial mucosa epithelial cells (▴). (H) Except for some alveolar cells (↑), SNA staining is negative. C, F and I, following pre-treatment with neuraminidase, neither MAAII nor SNA shows positive reaction. This confirms the specificity of both lectin stains. Scale bar, 50 µm.

We examined the receptor binding specificity in the respiratory tract of guinea pigs by lectin histochemistry assay. Biotinylated *Maackia amurensis* lectin II (MAA II), and *Sambucus nigra* agglutinin (SNA) were used to detect SAα2,3-Gal and SAα2,6-Gal linked receptors, respectively. In the nasal tract, both the SAα2,3-Gal and SAα2,6-Gal receptors were widely expressed by stratified squamous epithelial cells, vascular endothelial cells and the epithelial cells of the serous gland (Fig. 3 A, B). In the trachea, both of SAα2,3-Gal and SAα2,6-Gal receptors were widely expressed by epithelial cells of the tracheal mucosa (Fig. 3 D, E). In the lungs, SAα2,3-Gal receptor was mainly expressed by the alveolar cells and the vascular endothelial cells and was also expressed by the bronchial mucosa epithelial cells, however, SAα2,6-Gal receptor was only rarely expressed in the alveolar cells (Fig. 3 G, H). Taken together, the data suggested that SAα2,3-Gal and SAα2,6-Gal receptors were present in the nasal and tracheal areas of the guinea pig. In contrast, SAα2,3-Gal was the dominant receptor type in the lung.

## Discussion

In the present study, we investigated the guinea pig as a mammalian model for the study of the replication and transmission characteristics of selected swine H1N1, H1N2, H3N2 and avian H9N2 influenza viruses. We compared these characteristics to those of pandemic (H1N1) 2009, and seasonal human H1N1 and H3N2 influenza viruses. Our results showed that all the tested swine and avian influenza viruses replicated effectively in guinea pig nasal tracts without prior adaptation and the replication of these viruses was restricted to the respiratory system. The replication characteristics of swine and avian influenza viruses were similar to those of pandemic (H1N1) 2009 and seasonal human H1N1, H3N2 influenza viruses in addition to those of other influenza viruses reported by previous studies [13], [19], [21]. Moreover, some swine and avian viruses (namely, Sw/GD/211/06 (H3N2), Sw/GD/968/06 (H3N2) and Qa/HK/G1/97 (H9N2) viruses) were cleared more slowly from the guinea pig nasal passages than pandemic (H1N1) 2009 and seasonal human viruses. Histopathological analyses indicated that no significantly different pathological changes between human, swine and avian influenza viruses upon infection of the guinea pig. Of note, however, the histopathology after Qa/HK/G1/97 (H9N2) infection was the most severe of the viruses tested.

Pandemic (H1N1) 2009 influenza viruses possess effective human-to-human transmission ability and rapidly spread around the world. Similar to the transmissibility in humans, the transmission results in the present study in guinea pigs indicated that the 2009 pandemic virus can be passed from infected guinea pigs to all the uninfected contacts. Steel *et al.* has also demonstrate that transmission of a pandemic (H1N1) 2009 strain occurred with 100% efficiency in guinea pigs [11]. Two human seasonal viruses also showed transmission between guinea pigs. Additionally, Lamb *et al.* reported that during the 1918 epidemic, a parallel outbreak of pneumonial disease among their laboratory guinea pigs emerged [23]. Although sporadic instances of human infection with swine and avian influenza viruses occur, human-to-human transmission is rare. The transmission phenotype of swine and avian influenza viruses in guinea pigs was similar to those found in the human population. Tested swine and avian influenza viruses could replicate effectively in the respiratory system of guinea pigs, however, none could be transmitted from infected guinea pigs to contact animals. Taken together, the experimental transmission of human, swine and avian influenza viruses in guinea pigs correlated well with the transmissibility of these viruses in humans. These studies show the utility of the guinea pig model for not only the evaluation of the transmissibility of pandemic influenza strains, human seasonal viruses and H5N1 influenza viruses, but could also be used for low pathogenic swine and avian influenza viruses to help recognize the strains that possess potential human-to-human transmission ability.

The genetic composition of the 2009 pandemic H1N1 viruses probably resulted from the reassortment of recent North American H3N2 and H1N2 swine viruses (i.e., avian/human/swine “triple” reassortant viruses) with Eurasian avian-like H1N1 swine viruses [24]. In the present study, the shedding kinetics and tissue tropism of Sw/GD/1222/06 (H1N2, North American triple) and Sw/FJ/204/07 (H1N1, European avian-like swine) were similar to BJ/317/09 (H1N1, pandemic) and support the proposed origin of pandemic H1N1 influenza virus. However, the transmission phenotypes of these swine influenza viruses were significantly different from those of the 2009 pandemic strain. The 2009 pandemic influenza virus was able to transmit efficiently from inoculated guinea pigs to all exposed animals, while no transmission was observed for the swine influenza viruses. Thus, further studies are needed to determine how swine viruses that lack human-to-human transmissibility acquired efficient transmissibility resulting in current pandemic strains.

Many studies have demonstrated that the guinea pig was a good animal model for influenza transmission research, however, the reason for the susceptibility of guinea pigs to influenza infection is not yet clear. Tang *et al.* proposed that the nasal epithelial cells of guinea pigs support influenza virus growth along with the excessive nasal mucus secretions and these factors may contribute to the susceptibility of guinea pigs to droplet spread [13]. In the present study, sneezing was often observed in all the guinea pigs infected by the 2009 pandemic H1N1 influenza virus and this strain had the most efficient transmissibility in this study. Sneezing is a clinical sign of humans infected with influenza virus and is usually observed in ferrets infected with influenza virus, and is considered an important ability of a virus for the efficient respiratory droplet transmission to elicit symptoms that promote expulsion of virus from the host [25]. Although previous studies have not reported such clinical signs in influenza virus infected guinea pigs, sneezing in guinea pigs is usually measured in studies for antigen-induced rhinitis [26], [27]. Here, we propose that sneezing is perhaps another reason that guinea pigs should be considered an ideal influenza transmission model host.

Host receptor expression is one of the factors important for infection of host cells by influenza viruses. We examined the receptor specificity of the guinea pig respiratory system. The results obtained in this study by histochemistry were similar to those of Gao *et al.* found by fluorescence [21]. We found a widespread presence of both SAα2,6-Gal (SNA) and SAα2,3-Gal (MAA II) receptors in the nasal tract of guinea pigs, which suggests that these organs are potential targets for avian, swine and human influenza viruses. The results of animal experiments performed in this study, in addition to previous studies, demonstrated that the human, swine and avian influenza viruses replicated efficiently in this area of guinea pigs. Furthermore, virus replication at this site is likely to result in viruses that can be readily shed to infect other animals leading to the efficient transmission of influenza viruses in guinea pigs. The SAα2,6-Gal receptor is rarely detected in the upper respiratory tract of BALB/c mice, while SAα2,6-Gal is widespread on epithelial cells of the upper airways of humans [18], [28]. Therefore, compared with mice, guinea pigs that express both SAα2,3-Gal and SAα2,6-Gal in the upper respiratory tract reflect a similar anatomy to that of humans.

In summary, we found the high susceptibility of guinea pigs to swine and avian influenza viruses, the correlation of transmissibility of these animal influenza viruses in guinea pigs with those in humans and the similar receptor distribution in the respiratory system of guinea pigs to those of humans. Additionally, guinea pigs possess many other human-like characteristics, including airway innervation [14], bronchus-associated lymphoid tissue in the lung [15] and response to various mediators [29]. Thus, we suggested that guinea pig could be used as an appropriate animal model for evaluating the potential threat of swine and avian viruses for humans. Animal influenza viruses are repeatedly introduced into humans and frequent reassortment and evolution often occurs. To this end, the guinea pig model offers a useful animal model to assess circulating strains that could challenge human public health and an important tool for pandemic preparedness worldwide.

## Materials and Methods

### Ethics Statement

All animal research was approved by the Beijing Association for Science and Technology, the approve ID is SYXK (Beijing) 2007-0023, and complied with the guidelines of Beijing laboratory animal welfare and ethical of Beijing Administration Committee of Laboratory Animals.

### Viruses

The 2009 pandemic H1N1 virus, human seasonal H1N1 and H3N2, swine H1N1, H1N2 and H3N2, and avian H9N2 influenza viruses were used in this study (Table 1). Human seasonal H1N1 GD/41/06 and H3N2 JX/262/05 viruses were kindly provided by Dr. G. F. Gao (Institute of Microbiology, Chinese Academy of Sciences). The avian H9N2 influenza Qa/HK/G1/97 virus was kindly provided by Dr. H. Kida (Hokkaido University Graduate School of Veterinary Medicine). The pandemic (H1N1) 2009 BJ/317/09 viruses, swine and other avian influenza viruses were isolated by our laboratory in China, as previously described [30], [31].

### Replication of viruses in guinea pigs

Hartley strain female SPF/VAF guinea pigs weighing 300–350 g that were seronegative for influenza virus were used in these studies and were obtained from Vital River Laboratory Animal Technology. Zoletil 100 (tiletamine-zolazepam; Virbac S.A., Garros, France) (10–15 mg/kg) were used to anesthetize animals by intramuscular injection.

To investigate the replication of human, swine and avian influenza viruses in the guinea pig model, groups of 15 animals were anesthetized and inoculated intranasally with 10^6^ EID_50_ of tested virus in a 300 µL volume (150 µL on each side). On days 2, 4, 6, and 8 post-inoculation (p.i.), three animals from each group were euthanized and tracheas, lungs, hearts, livers, spleens, kidneys, brains, and colons were collected for virus titration in eggs. Nasal washes from the three remaining animals were collected on days 2, 4, 6, and 8 p.i., and titrated by EID_50_ assay. Nasal washing was performed by instilling a total of 1 mL of PBS containing antibiotics (Gibco) into the nostrils and collecting liquid runoff into a sterile Petri dish. These three animals were also observed for two weeks for body weight and temperature, signs of disease, and tested for seroconversion on day 21 p.i..

### Contact transmission in guinea pigs

Groups of three animals were intranasally inoculated with 10^6^ EID_50_ of tested virus and housed in a cage. At 24 h p.i., three naïve guinea pigs were placed in the same cage and co-housed in this way for a total of 20 days. On days 2, 4, 6, and 8 p.i., the nasal washes of the three inoculated animals and three contact animals were collected and titered for EID_50_ assay. All of the animals were humanely euthanized on day 21 p.i. and tested for seroconversion.

### Histopathology

On day 4 p.i., the nasal, trachea and lung specimens from inoculated guinea pigs were fixed in 10% neutral buffered formalin, routinely processed, and embedded in paraffin. Four-micrometer sections were stained with hematoxylin and eosin (HE).

### Detection of SAα2,3-Gal and SAα2,6-Gal receptors in guinea pig respiratory tissues

Biotinylated *Maackia amurensis* lectin II (MAA II), which is specific for α-2,3-linked sialic acid, and biotinylated *Sambucus Nigra* (Elderberry) bark lectin (SNA), specific for binding the α-2,6 linked sialic acid, were purchased from Vector Laboratories (Burlingame, CA, USA).

Two female Hartley SPF/VAF guinea pigs were euthanized and the nasal, trachea and lung tissues were fixed in 10% (w/v) neutral-buffered formalin for 24–48 h, and 4-µm thick paraffin-embedded tissue sections were made. Sections were stained with biotinylated sialic acid-specific lectins (MAA II or SNA) and the method of lectin histochemistry was performed as described by Yao *et al.* [32]. Briefly, paraffin-embedded serial sections were de-paraffinized and immersed in 3% hydrogen peroxide to eliminate endogenous peroxidase activity. A solution of 5% bovine serum albumin was used as the blocking agent to avoid non-specific staining. Sections were next incubated with SNA (0.625 g/mL) and MAA II (1.25 g/mL) at 4°C overnight. Serial sections of the same tissue were also incubated with PBS instead of lectin as negative controls. Optimal contrast between the specific labeling and the background was obtained with the use of a SABC kit (Dako, Carpinteria, CA, USA). Biotinylated lectin binding was visualized using a DAB (3,3′-diaminobenzidine-tetrahydrochloride) substrate chromogen kit (Zymed Labs, San Francisco, CA, USA) and slides were counterstained with hematoxylin.

### Neuraminidase pre-treatment

To confirm the specificity of the lectin stains, neuraminidase pre-treatment was performed. Because neuraminidase digests both α-2,3-linked and α-2,6-linked sialic acid residues, negative lectin staining after neuramidase pre-treatment would indicate that the lectin used was specific for detection of α-2,3-linked and α-2,6-linked sialic acids. Neuraminidase pre-treatment was performed as described by Yao *et al.* [32]. Briefly, the paraffin-embedded tissue sections were de-paraffinized and immersed in 3% hydrogen peroxide to eliminate endogenous peroxidase activity. Slides were covered with 12.5 U/µL neuraminidase solution (NEB, Ipswich, MA, USA) and incubated for 24 h at 37°C. Slides were washed three times with PBS and 5% BSA was used to block non-specific straining. Lectin staining was subsequently performed as described above. Additional negative controls were performed by using slides incubated with PBS instead of neuraminidase.

## Supporting Information

Figure S1
**Representative histopathological changes in HE stained nasal tissues (A–D), tracheas (E–H) and lungs (I–L) from guinea pigs on day 4 p.i..** (A) BJ/317/09 (H1N1) virus. Phlebectasia and congestion in submucosal capillaries and veins (↑). (B) Sw/GD/811/06 (H3N2) virus. Edema (↑) and congestion (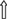). (C) Sw/GD/1222/06 (H1N2) virus. Disorganization of epithelial cells (↑); phlebectasia (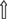) and hemorrhage in mucus (▴). (D) Qa/HK/G1/97 virus. Desquamation of the mucosal epithelium, inflammatory cells and erythrocytes adhering to the surface of mucosa (↑). (E) BJ/317/09 (H1N1) virus. Tracheal mucus membrane edema and thickening (↑). (F) Sw/GD/811/06 (H3N2) virus. Except for mild edema (↑), almost no lesions were present. (G) Sw/GD/1222/06 (H1N2) virus. Dropout of mucous epithelium in trachea (↑). (H) Qa/HK/G1/97 (H9N2) virus. Severe lesion of epithelial cells of tracheal mucosa (↑). (I) BJ/317/09 (H1N1) virus. Desquamation of epithelial cells of tunica mucosa bronchiorum in the bronchial lumen (↑) and a large number of inflammatory cell infiltrates around blood vessels, bronchi and pulmonary alveolus (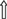). (J) Sw/GD/811/06 (H3N2) virus. Except for mild damage to the bronchioles (↑), almost no lesions were noticeable. (K) Sw/GD/1222/06 (H1N2) virus. A large number of inflammatory cell infiltrates around the blood vessels (↑). (L) Qa/HK/G1/97 (H9N2) virus. Disappearance of the normal structure of lung tissues; some bronchi were occluded with cellular debris, mucus and immune cells (↑); the whole lung tissues were filled with red blood cells, immune cells and inflammatory exudates. Scale bar = 50µm.(TIF)
