# Supplementary figures and images for: Guinea Pig Model for Evaluating the Potential Public Health Risk of Swine and Avian Influenza Viruses
Source: PLoS One. 2010 Nov 23;5(11):e15537. doi: 10.1371/journal.pone.0015537 (PMC2990763; doi:10.1371/journal.pone.0015537)

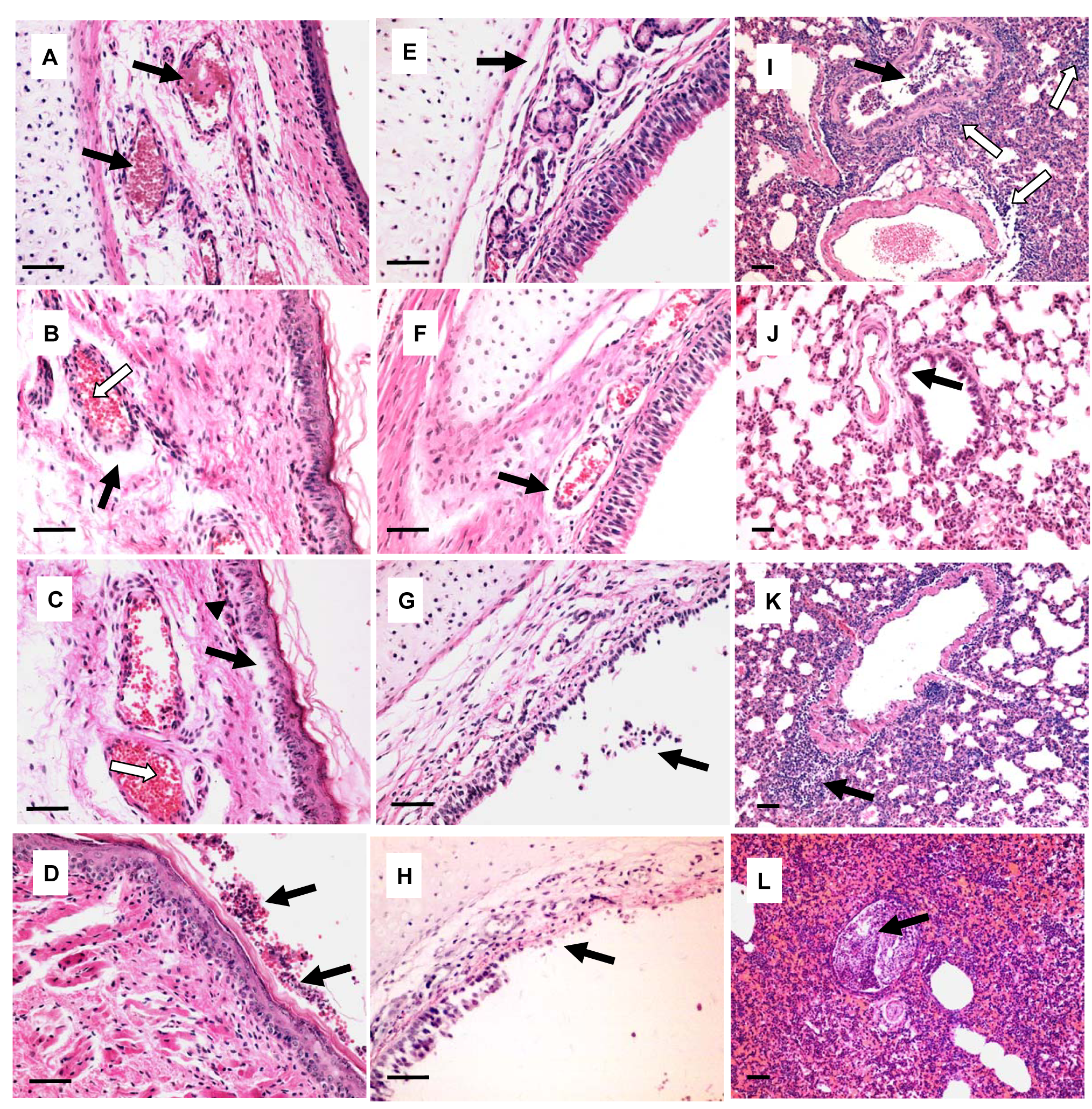

Supplement: Figure S1 — Representative histopathological changes in HE stained nasal tissues (A–D), tracheas (E–H) and lungs (I–L) from guinea pigs on day 4 p.i.. (A) BJ/317/09 (H1N1) virus. Phlebectasia and congestion in submucosal capillaries and veins (↑). (B) Sw/GD/811/06 (H3N2) virus. Edema (↑) and congestion (). (C) Sw/GD/1222/06 (H1N2) virus. Disorganization of epithelial cells (↑); phlebectasia () and hemorrhage in mucus (▴). (D) Qa/HK/G1/97 virus. Desquamation of the mucosal epithelium, inflammatory cells and erythrocytes adhering to the surface of mucosa (↑). (E) BJ/317/09 (H1N1) virus. Tracheal mucus membrane edema and thickening (↑). (F) Sw/GD/811/06 (H3N2) virus. Except for mild edema (↑), almost no lesions were present. (G) Sw/GD/1222/06 (H1N2) virus. Dropout of mucous epithelium in trachea (↑). (H) Qa/HK/G1/97 (H9N2) virus. Severe lesion of epithelial cells of tracheal mucosa (↑). (I) BJ/317/09 (H1N1) virus. Desquamation of epithelial cells of tunica mucosa bronchiorum in the bronchial lumen (↑) and a large number of inflammatory cell infiltrates around blood vessels, bronchi and pulmonary alveolus (). (J) Sw/GD/811/06 (H3N2) virus. Except for mild damage to the bronchioles (↑), almost no lesions were noticeable. (K) Sw/GD/1222/06 (H1N2) virus. A large number of inflammatory cell infiltrates around the blood vessels (↑). (L) Qa/HK/G1/97 (H9N2) virus. Disappearance of the normal structure of lung tissues; some bronchi were occluded with cellular debris, mucus and immune cells (↑); the whole lung tissues were filled with red blood cells, immune cells and inflammatory exudates. Scale bar = 50µm. (TIF) [file pone.0015537.s001.tif]
